# Safety of drotrecogin alfa (activated) treatment in patients with severe sepsis on renal replacement therapy without additional anticoagulation

**DOI:** 10.1186/cc9544

**Published:** 2011-03-11

**Authors:** L Mirea, I Luca Vasiliu, R Ungureanu, A Balanescu, I Grintescu

**Affiliations:** 1 Clinical Emergency Hospital, Bucharest, Romania

## Introduction

Patients with sepsis-induced acute renal failure on continuous renal replacement therapy (CRRT), who receive heparin, may be at higher risk of bleeding when drotrecogin alfa activated (DAA) is administered in addition to standard anticoagulation, especially surgical patients. There are some previous observations that no additional anticoagulation is necessary during simultaneous DAA infusion and CRRT. The aim of this study was to evaluate the safety of CRRT during DAA infusion without additional anticoagulant therapy.

## Methods

An observational, prospective study was conducted in an adult ICU. Sixteen surgical patients with severe sepsis on CRRT were divided into two groups: group A (eight patients) with DAA infusion, group B (eight patients) without DAA infusion. Baseline demographics, APACHE II score, serious bleeding events, and in-hospital mortality were reported. CRRT was performed using the Multifiltrate^® ^system, heparin-free continuous venovenous hemodialysis mode in group A. After the completion of the DAA infusion, intravenous standard heparin was administered for the remaining time on hemofiltration. In group B concomitant heparin was administered as necessary to achieve an aPTT of approximately 60 seconds.

## Results

The mean filter survival time (defined as the time until the circuit clotted) was 30 hours on DAA infusion versus 22 hours after DAA infusion in group A and 19.6 hours in group B. All survivors had recovery of dialysis-free renal function. The mean APACHE II score was 31.25 in group A and 22.12 in group B. Hospital mortality was 50% in group A (4/8) and 37.5% in group B (3/8); no mortality was attributed to bleeding. One case of severe thrombocytopenia was recorded with premature interruption of DAA infusion. The need for transfusion of blood and blood products infusion was compared (61% during DAA infusion vs. 52% after DAA infusion; 55% in group B); no serious bleeding event in both groups.

## Conclusions

The use of DAA in patients with severe sepsis requiring RRT is safe and is not associated with an increased of major bleeding events. No additional anticoagulation is necessary during simultaneous DAA infusion and CRRT.
